# Does Hypothyroidism Affect Gastrointestinal Motility?

**DOI:** 10.1155/2009/529802

**Published:** 2010-03-07

**Authors:** Olga Yaylali, Suna Kirac, Mustafa Yilmaz, Fulya Akin, Dogangun Yuksel, Nese Demirkan, Beyza Akdag

**Affiliations:** ^1^Department of Nuclear Medicine, Faculty of Medicine, Pamukkale University, Doktorlar Caddesi no. 42, Denizli 20100, Turkey; ^2^Division of Gastroenterology, Faculty of Medicine, Pamukkale University Hospital, Kınıklı, Denizli 20020, Turkey; ^3^Division of Endocrinology, Faculty of Medicine, Pamukkale University Hospital, Kınıklı, Denizli 20020, Turkey; ^4^Department of Pathology, Faculty of Medicine, Pamukkale University Hospital, Kınıklı, Denizli 20020, Turkey; ^5^Department of Biostatistics, Faculty of Medicine, Pamukkale University Hospital, Kınıklı, Denizli 20020, Turkey

## Abstract

*Background*. Gastrointestinal motility and serum thyroid hormone levels are closely related. Our aim was to analyze whether there is a disorder in esophagogastric motor functions as a result of hypothyroidism. *Materials and Methods*. The study group included 30 females (mean age ± SE 45.17 ± 2.07 years) with primary hypothyroidism and 10 healthy females (mean age ± SE 39.40 ± 3.95 years). All cases underwent esophagogastric endoscopy and scintigraphy. For esophageal scintigraphy, dynamic imaging of esophagus motility protocol, and for gastric emptying scintigraphy, anterior static gastric images were acquired. *Results*. The mean esophageal transit time (52.56 ± 4.07 sec for patients; 24.30 ± 5.88 sec for controls; *P* = .02) and gastric emptying time (49.06 ± 4.29 min for the hypothyroid group; 30.4 ± 4.74 min for the control group; *P* = .01) were markedly increased in cases of hypothyroidism. *Conclusion*. Hypothyroidism prominently reduces esophageal and gastric motor activity and can cause gastrointestinal dysfunction.

## 1. Introduction

There have been reports of disorders of motility and transport functions in the digestive system resulting from hypothyroidism [1, 2]. A reduction in the motor activity of stomach, small intestine, and colon has been reported in previous studies. Delayed intestinal transit time has been reported for hypothyroid patients, although normal gastric emptying time and normal intestinal transit time have also been reported for sufferers of thyroid disorders [1–7]. There have been a few esophageal motility and gastric emptying rate studies performed, but the pathophysiology of changes in the motor activity of digestive system observed in hypothyroid patients has not yet been determined [2, 7]. The most probable pathological reason is the intestinal edema due to mucopolysaccharide accumulation in gastrointestinal tissue, especially hyaluronic acid [7]. Thus, the objective measurement of gastric emptying time is crucial and gastric emptying studies using radionuclides, a noninvasive physiological method, are the accepted gold standard [8]. Hypothyroid patients also suffer from a feeling of distress in the cervical region similar to dysphagia. Radiographic methods have previously been used to evaluate esophageal function disorders, but the absorbed radiation doses with these methods are too high. A manometry study, an invasive method, is accepted as the gold standard to evaluate esophageal dysmotility by many researchers [9, 10]. Since esophageal motility studies with radionuclides have come into practice, researchers have been able to evaluate esophageal motor function noninvasively, practically, and with a minimal radiation dose.

Our aim was to evaluate whether upper gastrointestinal system motility is affected in hypothyroid patients by acquiring and evaluating esophageal and gastric scintigraphies.

## 2. Materials and Methods

### 2.1. Cases

Thirty female patients admitted to endocrinology with a diagnosis of primary hypothyroidism and having no other systemic disease, except minor dyspeptic complaints, were enrolled. These patients (mean ± SE: 45.17 ± 2.07 years) did not smoke, or drink alcohol, or take any medication that would affect esophagogastric motility. Control group consisted of 10 healthy females (mean ± SE: 39.40 ± 3.95 years). The period between the beginning of the hypothyroid symptoms and diagnosis was 2–4 months. The study received permission from the Faculty Ethics Committee, and an informed consent form was obtained for all patients before tests were performed.

Thyroid hormones (FT3, FT4), thyroid stimulating hormone (TSH) levels, vitamin B12 (Vit B12) levels, complete blood counts (hemoglobulin, hematocrit, white blood cells, red blood cells, and platelets), sedimentation rate, and routine biochemical blood tests (serum creatinine, urea, uric acid, electrolytes, albumin, total protein, liver enzymes, triglycerides, cholesterol, and glucose) were measured for all cases. Then, all cases underwent gastroesophageal motility scintigraphy.

### 2.2. Scintigraphy Studies

All scintigraphic images were acquired with a circular CamStar AC/T gamma camera (GE Healthcare, Milwaukee, WI, USA) using a LEAP. For the esophageal motility scintigraphy, the patient fasted for 6 hours. Then, the patient sat up, faced the front of the camera, and was instructed to hold in her mouth a solution of 15 cc of water including 18 MBq of ^99m^Tc nanocolloid (Nanocis – Tc-99m colloidal rhenium sulphide injection [nanocolloid]; CIS Bio International; Cedex, France). Next, simultaneous to acquiring the anterior esophageal dynamic images, the patient was instructed to swallow all of the solution within 1 minute by swallowing once every 5 to 10 seconds. This standard protocol has been applied to all patients. Dynamic images were acquired over 60 seconds at a rate of 1 frame/sec onto a 64 × 64 matrix. After imaging, a cine-image of the esophagus transition was observed on the processing unit. A region of interest (ROI) was drawn on the esophagus, excluding the stomach's fundus. Esophageal emptying (EE) and transit time (ETT) were automatically calculated by using formulae of ETT (sec): Time period between the starting point of detected radioactivity and 10% radioactivity remaining after the radioactivity peak, and, EE (%): (Max count-number of counts at 10 sec after max counts)/(Max counts-mean residual counts preceding initial swallow).

The gastric emptying time scintigraphy was acquired within 2 days after the esophageal scintigraphy and after a night of fasting (minimum 8 h). To prevent the probable hormonal effects of ovulation on gastrointestinal motility, cases were analyzed within the first 10 days of their menstrual cycle. The study was conducted by asking the patients to eat 230 cc of semisolid food containing 37 MBq Tc-99m nanocolloid. The food, which consisted of 80 cc milk and 150 gr corn cereal for a total of 500 kcal, had both a dense, solid component and a less dense, liquid component. Immediately following consumption, the patient was supine. Static anterior gastric images were acquired every 15 minutes for up to a maximum of 120 minutes; acquisitions were 1 minute in duration. ROIs were drawn on the gastric region for all images, and gastric emptying time activity curves and gastric emptying half-times (T_50_) were calculated automatically.

### 2.3. Endoscopy and Pathological Analysis

All patients underwent gastroesophageal endoscopy and gastric mucosa biopsies. All gastric biopsy materials were examined for probable mucopolysaccharide accumulation in gastric mucosa using periodic acid shift (PAS) dye [11, 12]. Gastric mucosae of the patients were categorized into three groups based on endoscopic and histopathological analyses: normal gastric mucosa (*n* = 1), acute erosive gastritis (*n* = 12), and atrophic gastritis (*n* = 17).

### 2.4. Statistical Analysis

The descriptive statistics were given as median and minimum-maximum values. Data were analyzed by using Mann-Whitney U-test and Spearman correlation coefficient. The statistical significance was set at *P* < .05. All analyses were performed with the SPSS (version 10.0) statistical package program.

## 3. Results

Routine CBC and biochemical values of the patients were within normal limits. For the hypothyroid group, the median TSH value measured was 9.6 (0.56–100) *μ*IU/mL (normal range: 0.27–4.2 *μ*IU/mL), and it was significantly higher than that of control groups (Table 1). The median Vit B12 value was 200 (140–683) pg/mL (normal range: 193–982 pg/mL). Though the mean Vit B12 value for the hypothyroid group was within normal limits, it was significantly lower than that of control group (Table 1). No significant correlation was found between TSH (*R* = −0.115, *P* = .437 for ETT; *R* = 0.283, *P* = .130 for T_50_) and Vit B12 level (*R* = −0.115, *P* = .545 for ETT; *R* = 0.032, *P* = .868 for T_50_) and the esophagogastric motor activity of hypothyroid patients.

**Figure 1:**
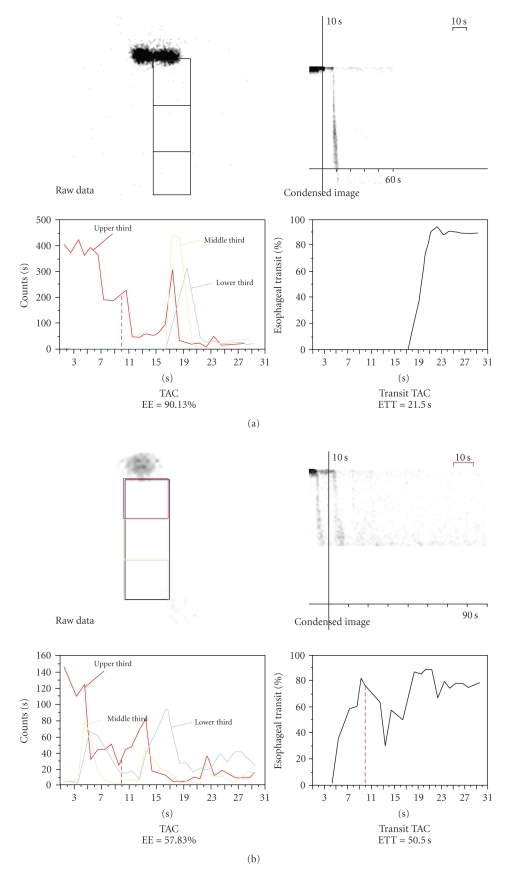
(a) The normal esophageal emptying (90.13%) and esophageal transit time (21.50 sec) in a healthy case of control group. (b) The delayed esophageal emptying (57.83%) and the extended esophageal transit time (50.50 sec) in a hypothyroid patient.

**Figure 2:**
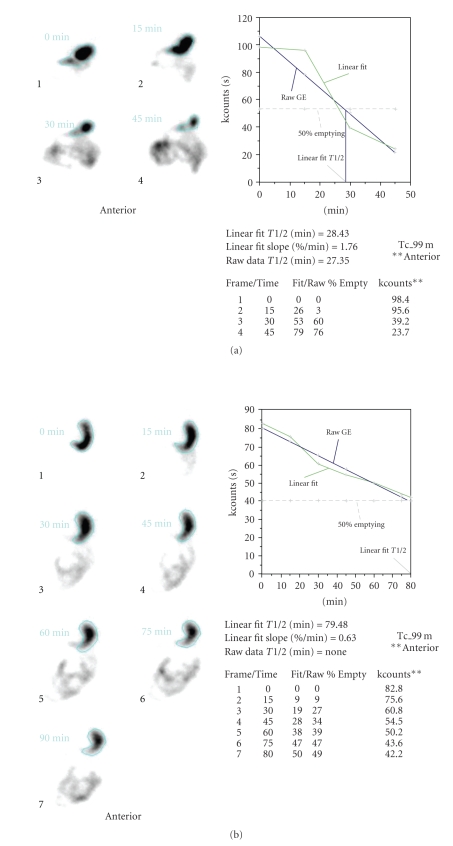
(a) The normal gastric emptying half-time (T50: 28.43 min) in a healthy case of control group. (b) The delayed gastric emptying half-time (T50: 79.48 min) in a hypothyroid patient.

All scintigraphic parameters of esophagogastric system were compared between the hypothyroid and control group in our study (Table 1). Comparing the groups, ETT and T_50_ values were found to be significantly higher, and the EE value was significantly lower (Figures 1(a), 1(b), 2(a), and 2(b)) in hypothyroid patients (*P* < .05). There was no significant difference between the gastroesophageal motility parameters of the patients suffering from atrophic or erosive gastritis. Also, no mucopolysaccharide accumulation in the gastric mucosa was observed in any of the samples examined using PAS dye.

## 4. Discussion

Gastrointestinal system disorders are ignored in hypothyroidism because of certain systemic symptoms of cardiovascular, neuromuscular, and ocular disorders with thyroid dysfunctions [13]. Changes in the motor activity of the digestive system may result in gastric distension and constipation in hypothyroidism [7, 14]. There are a limited number of studies about gastroesophageal function analysis in people with thyroid disorders compared to intestinal motility analysis. It has been found that hypothyroid patients show significant reduction in gastric emptying [1, 2, 7, 15] and that no relationship exists between thyroid hormone deficiency, gastric excretion, and acid secretion [16]. Our patients had primary hypothyroidism, without any systemic disorder, for 2–4 months, and they were all suffering from minor dyspeptic problems. None of them suffered from severe gastrointestinal system complaints such as nausea, vomiting, abdominal pain, or constipation. Comparing the esophagogastric scintigraphic parameters in our patients with those of healthy cases, we found a significant reduction in both gastric and esophageal motor functions. These results were similar with many published studies showing that hypothyroidism affects gastrointestinal system motility [1, 2, 7, 13, 15]. 

 It has been reported that the most physiologically relevant method to assess gastric motility is gastric emptying scintigraphy following a semisolid meal [1, 8, 15, 17, 18]. Therefore, most nuclear medicine departments prefer this method to evaluate gastric motility [8, 15]. Tc-99m colloid components used with semisolid food mark the solid phase of the meal more stably than the liquid phase, so the acquired data reflect solid phase measurements more than the liquid ones [8, 19]. Some data indicate that gastric emptying time is normal in liquid gastric emptying studies in healthy and short-term hypothyroid patients [5]. It has also been reported that euthyroid patients who have been treated for hypothyroidism no longer suffer from a delay in gastric emptying [6]. Unfortunately, there are a limited number of studies that examine gastric emptying time with solid food in hypothyroid patients [8]. Couturier et al. have stated that liquid parameters can be used if the solid phase takes too long to be measured [8]. We calculated the gastric emptying half-time on sequentially obtained images for 60–120 minutes for all patients and had no problems evaluating the gastric emptying function. The gastric emptying values in our healthy cases were found to be within normal limits defined by our clinic gastric emptying studies, so we believe that our results that gastric emptying time determined using semisolid food labeled with Tc-99m nanocolloid are reliable.

Reviewing the data of various scintigraphic studies where normal rates and pathologies of esophagus are evaluated, it has been reported that scintigraphic studies where the patients sit upright and swallow multiple times are much more reliable and physiologically relevant. Values of residual radioactivity in the esophagus between 13.1%–19.8% have been accepted as normal [19–21], and these are similar to the values (mean value is 19.8%) obtained from our control group. In hypothyroid patients, the significant elevation in residual esophageal radioactivity (mean excreted radioactivity is 64.1%; mean residual radioactivity is 35.9%) supports our hypothesis that there is deceleration in esophageal motor activity due to thyroid hormone deficiency. Unfortunately, we were unable to benchmark our data on esophageal motor activity because of the insufficiency of published data on esophageal motility. More research, thus, needs to be conducted in the field of esophageal motility disorders.

The reports that depletion of thyroid hormones inhibits the secretory functions of the digestive tract are fairly concordant [22, 23]. Hypothyroidism affects the entire gastrointestinal system and causes hypomotility [1, 7, 15]. This finding has been supported by data showing that gastrointestinal transition time is within normal limits in euthyroid cases following treatment of hypothyroidism [7]. However, it was previously indicated that there is no direct correlation between the abnormalities in gastrointestinal system kinetics and the level of hypothyroidism [1]. Similarly, in our study, no significant correlation has been found between esophagogastric motility and TSH values. We suspect that symptoms of gastrointestinal system dysmotility, such as dyspepsia, can occur with mild or severe hypothyroidism. Therefore, symptoms should be assessed in these patients.

 It is known that thyroid autoantibodies arising from autoimmune thyroid diseases may lead to atrophic gastritis and that mucosal atrophy of the fundus can occur without symptoms. Frequently, there is autoimmune pathogenesis, and it may even develop into pernicious anemia and gastric malignancy in the following years [24, 25]. In the case of hypothyroidism, mucinous material (mucopolysaccharide, hyaluronic acid, etc.) may accumulate in gastrointestinal system mucosa, which may lead to dysmotility. This accumulation can be observed via pathological analysis. However, if there is short-term hypothyroidism, no accumulation may be observed in gastrointestinal system mucosa [26, 27]. We examined all patients via gastroesophageal endoscopy and evaluated them histopathologically. The aim was to determine if there was atrophic gastritis and/or other probable gastric pathologies (e.g., mucopolysaccharide accumulation) and whether there were accumulations and whether they affected esophagogastric motor parameters. We did not observe any mucopolysaccharide accumulation in our patient group, whose complaints did not last longer than 4 months. No significant difference was found between the gastroesophageal scintigraphical motor parameters of the patients with atrophic gastritis and the ones with erosive gastritis. Therefore, we think that there is no direct correlation between the delayed gastric emptying and gastric mucosal pathology. Although there is no definite explanation of the pathogenesis of gastrointestinal system motor hypoactivity, a number of theories have been put forward: deceleration in myoelectric activity, autonomous neuropathy, intestinal edema, ischemia, reduction in *β* adrenergic receptors, and changes in peptic hormone metabolism [15, 27]. Nevertheless, there are a limited number of studies that have analyzed gastric myoelectric activity disorders in hypothyroid patients, so more pathophysiological studies should be conducted in order to clarify this topic. We are in the process of evaluating gastroesophageal motor functions after the treatment of hypothyroidism in this group of patients. 

 There are some possible limitations of our study which are the limited number of cases and no incidental diagnosis of hypothyroidism.

## 5. Conclusion

Hypothyroidism prominently decreases gastroesophageal motility, and as such, thyroid functions should be evaluated in patients admitted with complaint of dyspepsia. Gastroesophageal scintigraphies are noninvasive, simple, physiological methods to use for evaluating esophagogastric motility and can be helpful in selecting treatment regimes for hypothyroidism.

## Figures and Tables

**Table 1:** The scintigraphic parameters of esophagogastric system and statistical analysis in hypothyroid patients and control group.

Group	Age	TSH	Vit B12	Esophageal	Esophageal	Gastric
	(year)	(microIU/mL)	(pg/ mL)	emptying (%)	Transit time (sec)	emptying half-time (min)
Hypothyroid (*n* = 30)						
Median	46.50	9.6	200	76	60	44.50
(min-max)	(19–67)	(0.56–100)	(140–683)	(1–93)	(3–90)	(50–120)
Control (*n* = 10)						
Median	40.50	2.45	395	84.50	18.5	28
(min-max)	(22–56)	(1–3.5)	(250–605)	(54–93)	(5–58)	(12–60)
*P*	.182	.001	.0001	.045	.002	.010
